# Machine learning-optimized metabolic biomarker panel for precision screening of early-stage pancreatic cancer in new-onset diabetes

**DOI:** 10.3389/fendo.2025.1684608

**Published:** 2025-12-08

**Authors:** Weiliang Jiang, Zhiyuan Cheng, Rong Mu, Haoran Sun, Zihao Guo, Guo Yu, Dongyan Wang, Lijuan Yang

**Affiliations:** 1 Department of Gastroenterology, Shanghai General Hospital, Shanghai Jiao Tong University School of Medicine, Shanghai, China; 2 Department of Gastroenterology, Gongli Hospital of Shanghai Pudong New Area, Shanghai, China; 3 School of Gongli Hospital Medical Technology, University of Shanghai for Science and Technology, Shanghai, China

**Keywords:** metabolic biomarkers, pancreatic ductal adenocarcinoma, new-onset diabetes, early detection, metabolomics, machine learning

## Abstract

**Introduction:**

New-onset diabetes (NOD) represents a high-risk population for pancreatic ductal adenocarcinoma (PDAC), yet effective early detection tools for this specific subgroup remain an unmet clinical need.

**Methods:**

We conducted a prospective serum metabolomic analysis using UHPLC-MS/MS in 133 NOD patients aged >65 years, including 60 with PDAC (PDAC+NOD) and 73 without (NOD). Multivariate analysis (OPLS-DA) and machine learning approaches were employed to identify and optimize a diagnostic metabolic biomarker panel. Model performance was evaluated using a hold-out validation set following TRIPOD-ML guidelines.

**Results:**

We identified 62 differentially expressed serum metabolites (P<0.05, FDR-corrected), primarily implicating branched-chain amino acid metabolism, bile acid biosynthesis, and sphingolipid signaling pathways. Notably, significant reductions in one-carbon metabolism-related metabolites (serine, glycine, homocysteine) were observed in PDAC+NOD patients. Feature selection yielded an optimized 5-metabolite panel comprising glycine, L-serine, L-methionine, L-homocysteine, and L-homocystine. This panel demonstrated high diagnostic accuracy with an AUC of 0.853 (95% CI: 0.786-0.920) and 75.0% accuracy in distinguishing PDAC+NOD from NOD patients.

**Discussion:**

Our study establishes a foundational metabolic biomarker strategy for precision screening of early-stage PDAC in NOD populations. The dysregulated one-carbon metabolites provide novel mechanistic insights into PDAC pathogenesis and offer actionable targets for clinical assay development. Future validation in multi-center cohorts is warranted to confirm clinical utility.

## 1 Introduction

Pancreatic ductal adenocarcinoma (PDAC) remains one of the most lethal malignancies, with a five-year survival rate below 10%, largely attributable to late-stage diagnosis and frequent metastatic presentation (1–3). While surgical resection offers the only potential for cure, over 80% of patients present with inoperable, locally advanced, or metastatic disease (3, 4). This underscores the critical need for early detection strategies. The challenge is compounded by the difficulty in accurately assessing disease stage, such as predicting lymph node metastasis, even after tumor identification (5).

The clinical standard serum biomarker, CA19-9, has significant limitations for early detection, including poor sensitivity for early-stage PDAC, lack of expression in Lewis antigen-negative individuals, and non-specific elevation in benign conditions (6–8). Consequently, developing novel biomarkers with superior sensitivity and specificity is imperative.

Extensive efforts have focused on identifying blood-based biomarkers (9–15).Recent research explores diverse avenues, including serum exosomal microRNAs for distinguishing metastatic disease (5), and integrated metabolite-protein models for early detection (16). Metabolomic signatures (17) and radiomics approaches for tumor staging and lymph node prediction also show promise (18, 19). However, the low population incidence of PDAC makes validating the early screening potential of these markers challenging and renders general population screening economically unfeasible, necessitating a focus on high-risk cohorts.

New-onset diabetes (NOD) has emerged as a key risk factor and potential precursor to PDAC, providing a critical window for early detection. Up to 80% of PDAC patients develop hyperglycemia or diabetes within three years preceding their cancer diagnosis (20, 21). The PDAC risk is markedly elevated in individuals with recent-onset NOD (<1 year), demonstrating a 5.4- to 8-fold increased risk compared to the general population, substantially higher than in long-standing diabetes (20, 22, 23). This positions NOD as a prime high-risk group for targeted screening, offering a strategic and cost-effective opportunity to improve early diagnosis.

Prior studies have analyzed serum metabolomics (24) and proteomics (25) in NOD patients with and without PDAC, identifying potential biomarkers. However, these findings lack robust validation, and no biomarker has yet achieved clinical utility for early PDAC detection in this high-risk population.

To address this gap, we employed ultra-high-performance liquid chromatography tandem mass spectrometry (UHPLC-MS/MS) to conduct comprehensive serum metabolomic profiling, comparing NOD patients with and without PDAC. Using multivariate analysis and machine learning, we aimed to identify and validate a specific metabolic biomarker panel for the early detection of PDAC within this high-risk NOD cohort.

## 2 Materials and methods

### 2.1 Study design and participants

We conducted a prospective, single-center diagnostic model-development study in accordance with the TRIPOD-ML guidelines. Participants were consecutively recruited from Shanghai General Hospital, Shanghai Jiao Tong University School of Medicine, between March 2021 and November 2023.

Initial screening identified 210 eligible patients. After implementing the inclusion and exclusion criteria (detailed below; see **Supplementary Figure 1** for participant flow diagram), 133 participants were included in the final analytical cohort: 60 with PDAC and NOD (PDAC+NOD group) and 73 with NOD alone (NOD group). Complete data were available for all key metabolic biomarkers and clinical variables required for model development. All participants provided written informed consent, and the study protocol received approval from the Medical Ethics Committee of Shanghai General Hospital (Approval No: 2021KY095).

Inclusion Criteria comprised: (1) ECOG performance status 0–2 with estimated survival ≥12 weeks; (2) Fasting blood glucose levels meeting diagnostic criteria for glycemically-defined NOD; (3) Age >65 years; (4) NOD diagnosis duration ≤3 years; (5) For PDAC+NOD group: histopathological confirmation of pancreatic ductal adenocarcinoma or clinical diagnosis supported by comprehensive imaging assessment.

Exclusion Criteria included: (1) Significant comorbidities affecting major organ systems; (2) Active infectious diseases; (3) Metastatic cancer or additional primary malignancies; (4) Known high-risk predisposition for pancreatic cancer (e.g., familial pancreatic cancer syndromes); (5) Incomplete information.

### 2.2 Clinical and laboratory assessment

Venous blood samples were collected following a 12-hour overnight fast for untargeted metabolomic profiling. Comprehensive clinical data were systematically collected, including demographic characteristics, medical history, and laboratory parameters. The laboratory assessment encompassed diabetes duration, fasting glucose, hepatic function markers (total bilirubin, direct bilirubin, total bile acid, albumin), lipid profiles (cholesterol, triglycerides), and established tumor markers (CEA, CA19-9). Pathological staging was determined according to the American Joint Committee on Cancer (AJCC) 8th edition guidelines.

### 2.3 Chemicals and reagents

HPLC−grade methanol and acetonitrile (Fisher Chemicals), formic acid (Merck), 2−chloro−DL−phenylalanine (Merck), and one−carbon metabolism standards (Shanghai Yuanye Bio−Technology, purity >98%) were used.

### 2.4 Sample preparation for metabolomics study

Serum samples (100 *μL*) were mixed with 400 *μL* methanol and 5 *μL* fenclonine (internal standard), vortexed, and centrifuged (12,000 r/min, 4°C, 15 min). The supernatant (200 *μL*) was analyzed using UPLC coupled to an Orbitrap Elite mass spectrometer (parameters in **Supplementary Materials**).

### 2.5 Data processing and biomarker screening

Raw LC-MS data were processed using Compound Discoverer™ (version 3.0) for peak detection, retention time alignment, and metabolite identification. Data normalization was performed by total area scaling followed by probabilistic quotient normalization (PQN) using quality control (QC) samples. Multivariate statistical analyses, including PCA and OPLS-DA, were conducted in SIMCA-P (version 14.1), with metabolites exhibiting VIP scores >1.0 selected as candidate biomarkers. QC samples were analyzed every six experimental samples, meeting predefined acceptance criteria (retention time drift <0.1 min; peak area CV <15%; mass accuracy <5 ppm). ComBat correction was applied to address potential batch effects, demonstrating minimal impact on model performance (ΔAUC = 0.008).

### 2.6 Quantitative analysis of one−carbon metabolites

Targeted quantification of one−carbon metabolites was performed using UPLC−MS/MS (Waters Acquity; AB SCIEX 6500). Detailed instrument parameters are provided in **Supplementary Table 1**.

### 2.7 Sample preparation for targeted metabolite analysis

Serum (150 *μL*) was treated with 50 *μL* DTT (15 mg/mL), vortexed, mixed with 800 *μL* methanol, and centrifuged (10,000 rpm, 4°C, 15 min). The supernatant was dried under nitrogen and reconstituted in 75 *μL* DTT solution (10 *μ*g/mL) prior to analysis.

### 2.8 Statistical analysis

Data were analyzed in SPSS 25.0. Continuous variables (mean ± SD were compared using t-tests or Wilcoxon tests; categorical variables (percentages) were assessed with Chi-square tests. Receiver operating characteristic (ROC) analysis and logistic regression were applied, with *p* < 0.05 considered significant.

### 2.9 Machine learning model development and validation

To develop a robust diagnostic model for discriminating between PDAC+NOD and NOD patients, a comprehensive machine learning pipeline was implemented using Python 3.11.0 with the scikit-learn library (version 1.0.2). Initially, metabolite concentrations were log-transformed and standardized via z-score normalization. Missing values, which constituted less than 5% of the data, were imputed using the median value for the respective feature.

For feature selection, Recursive Feature Elimination with 5-fold stratified Cross-Validation (RFECV) was employed to identify the most informative and compact biomarker signature. This process yielded an optimal panel of five one-carbon metabolism metabolites: glycine, L-serine, L-methionine, L-homocysteine, and L-homocystine. Four distinct classification algorithms were subsequently evaluated: Gradient Boosting (GB), Random Forest (RF), a Support Vector Machine (SVM) with a radial basis function kernel, and regularized Logistic Regression (LR). Hyperparameter tuning for each algorithm was conducted using a grid search approach with 5-fold stratified cross-validation on the training data to ensure optimal model configuration.

Model training and validation were performed using a stratified 5-fold cross-validation scheme across the entire dataset to provide robust and unbiased performance estimates. The model’s diagnostic performance was quantified using the area under the receiver operating characteristic curve (AUC-ROC), accuracy, sensitivity, specificity, precision, and F1-score. To assess the incremental value of the 5-metabolite panel, the final GB model was compared against two baseline models: a univariate model using glycine alone and a multivariate model using the clinical variables of age, BMI, and diabetes duration. Statistical comparisons of AUCs were performed using DeLong’s test, supplemented by Net Reclassification Improvement (NRI) and Integrated Discrimination Improvement (IDI) to quantify the enhancement in classification accuracy.

Further, the model’s clinical utility was rigorously assessed. Model calibration was evaluated using calibration curves and the Brier score to measure the concordance between predicted probabilities and actual outcomes. Decision curve analysis (DCA) was performed to estimate the net benefit of using the model for clinical decision-making across a range of threshold probabilities. To contextualize the model’s performance for a real-world screening scenario, positive predictive value (PPV) and negative predictive value (NPV) were calculated based on PDAC prevalence rates of 0.5% and 1.0% reported in NOD populations. Finally, a series of sensitivity and subgroup analyses were conducted to test the model’s robustness across different normalization methods, age strata, BMI categories, and in patient cohorts defined by metabolite ratios, such as the glycine/serine ratio.

## 3 Results

### 3.1 Study cohort characteristics

The study included 133 participants (73 NOD, 60 PDAC+NOD) with comparable gender distribution. Significant intergroup differences were observed in diabetes duration, biliary obstruction, fasting glucose, total bilirubin, direct bilirubin, bile acid, albumin, triglyceride, and CEA (p < 0.05), while no significant differences were found in age, sex, BMI, cholesterol, hypertension, smoking, or CA19-9 (**Supplementary Table 2**).

### 3.2 Metabolic profiling and biomarker identification

**Figure 1:**
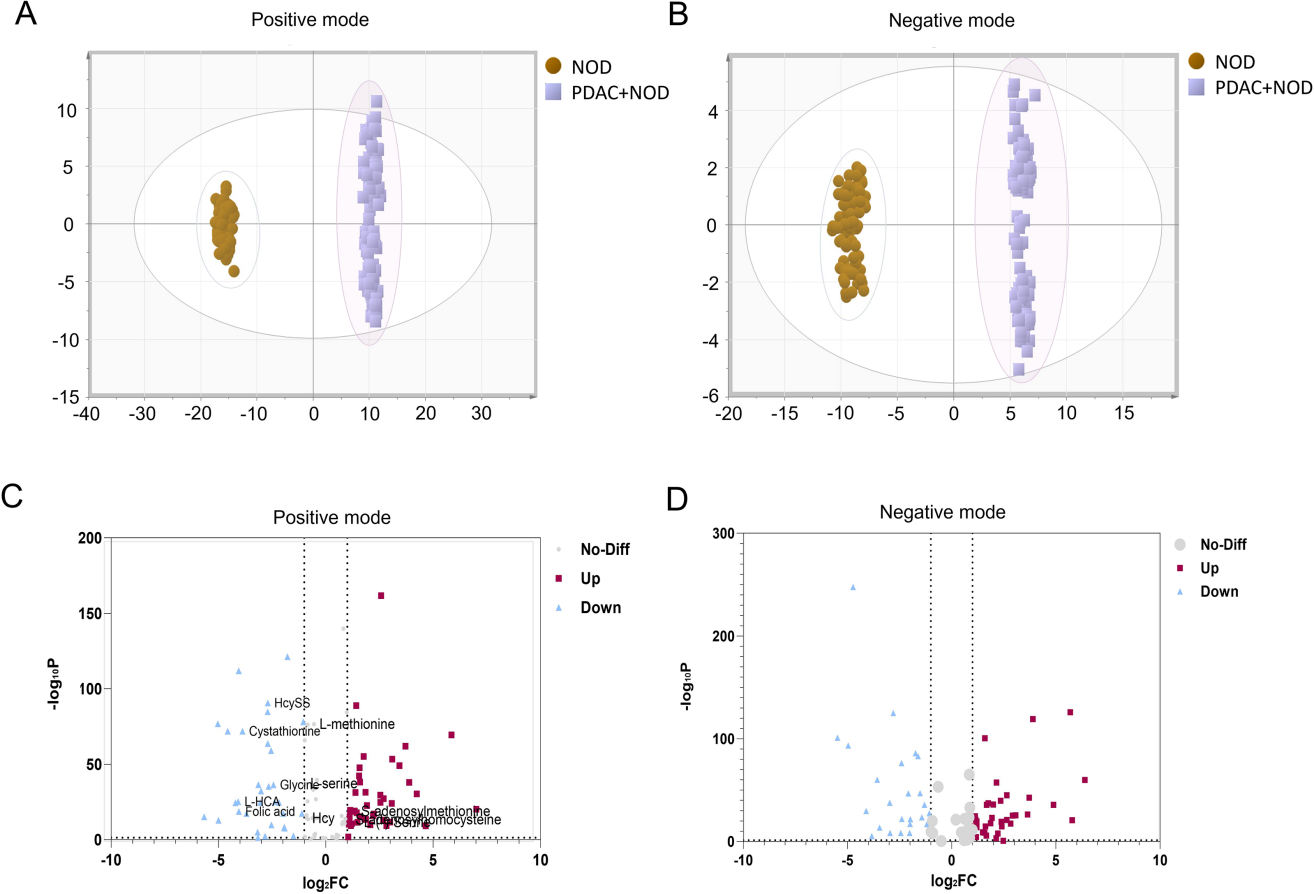
Discriminative serum metabolic profiling between NOD and PDAC+NOD groups. **(A)** PCA in positive mode, **(B)** PCA in negative mode, **(C)** Volcano plot of metabolites by univariate analysis in positive mode. **(D)** Volcano plot of metabolites by univariate analysis in negative mode.

**Figure 2:**
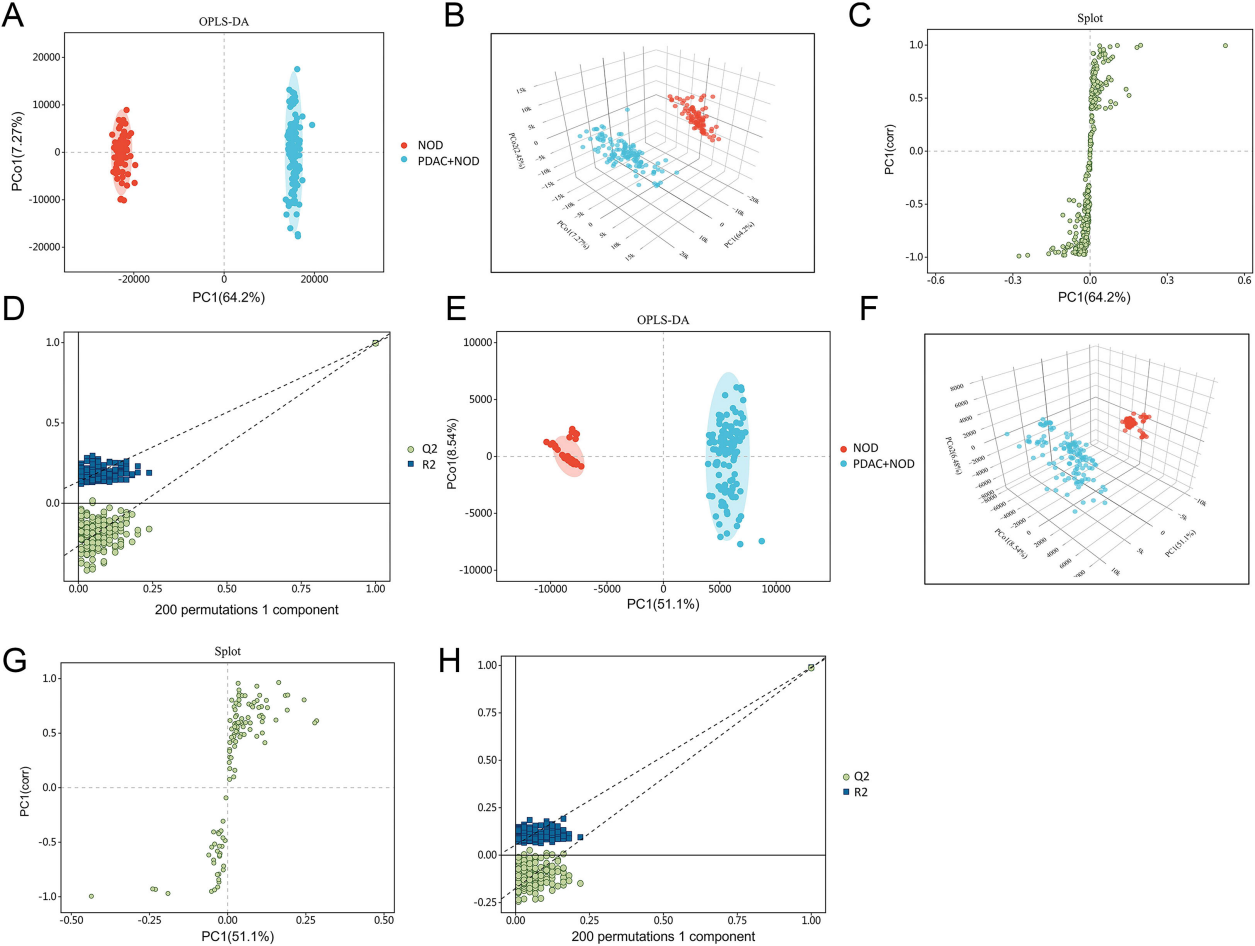
Multidimensional metabolic profiling by OPLS-DA. **(A)** OPLS-DA in positive mode, **(B)** OPLS-DA 3D plots in positive mode, **(C)** Splot in positive mode, **(D)** 200 permutations tests in positive mode, **(E)** OPLS-DA in negative mode, **(F)** OPLS-DA 3D plots in negative mode, **(G)** Splot in negative mode, **(H)** 200 permutations tests in negative mode.

**Figure 3:**
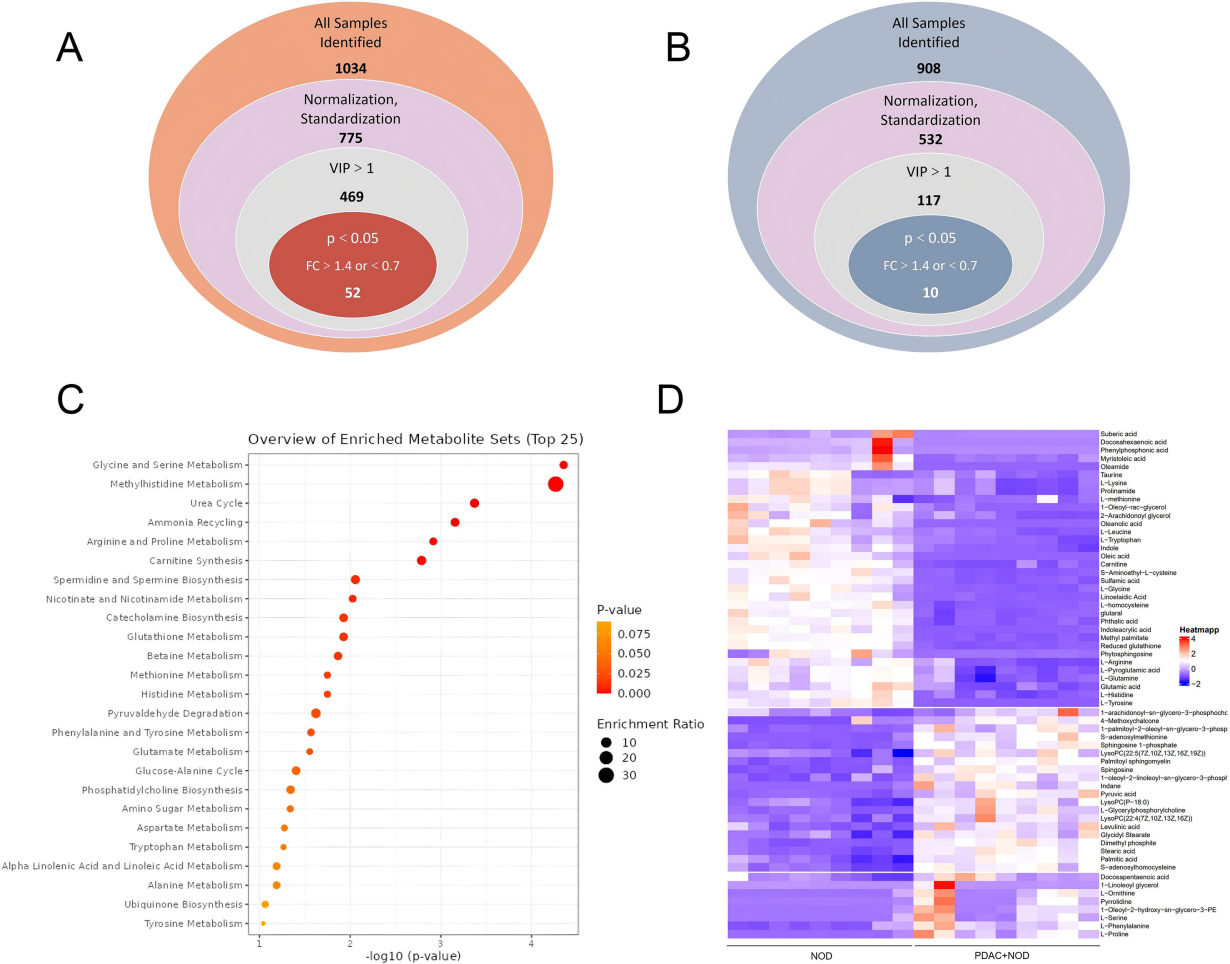
Identification, expression characterization, and pathway enrichment analysis of differential serum metabolites between NOD and PDAC+NOD Groups. **(A)** Venn diagram displaying the screening and constitution of altered metabolites in the NOD compared with PDAC + NOD in positive mode, **(B)** Venn diagram displaying the screening and constitution of altered metabolites in the NOD compared with PDAC + NOD in negative mode. **(C)** Expression heatmap of the 62 identified metabolites in the serum of NOD and PDAC+NOD groups. **(D)** Enriched metabolic pathways of differential metabolites. Pathway analysis results should be interpreted as exploratory findings.

Untargeted metabolomics revealed distinct separation between NOD and PDAC+NOD groups in both positive and negative ionization modes (**Figures 1**, **2**). We used Vip>1 and Fold change>1.4 or<0.7, P<0.05 as screening criteria, and finally identified a total of 62 significantly altered differential metabolites (**Figures 3A–C**; **Supplementary Table 3**). The pathway enrichment results showed glycine and serine metabolism, methyhistdine metabolism, arginine and proline metabolism, glutathione metabolism and methionine metabolism as most prominent (**Figure 3D**).

### 3.3 Analytical validation of metabolite quantification

Chromatographic separation and MS/MS parameters were systematically optimized to ensure precise metabolite detection. Representative chromatograms of standard solutions and serum samples (with internal standard) are provided in **Supplementary Figures 2**, **3**. Key analytical parameters—including regression equations, linear ranges, correlation coefficients (r² ≥ 0.9919), and lower limits of quantification (LLOQs)—are summarized in **Supplementary Table 4**. Comprehensive validation data covering precision, accuracy, matrix effects, and stability under various storage conditions are detailed in **Supplementary Tables 5**–**7**, collectively confirming the reliability and robustness of our quantitative methodology. Furthermore, all analytical batches consistently met the predefined acceptance criteria, as demonstrated in **Supplementary Figure 4**.

### 3.4 Content of one-carbon metabolite in NOD and PDAC+NOD group

**Figure 4:**
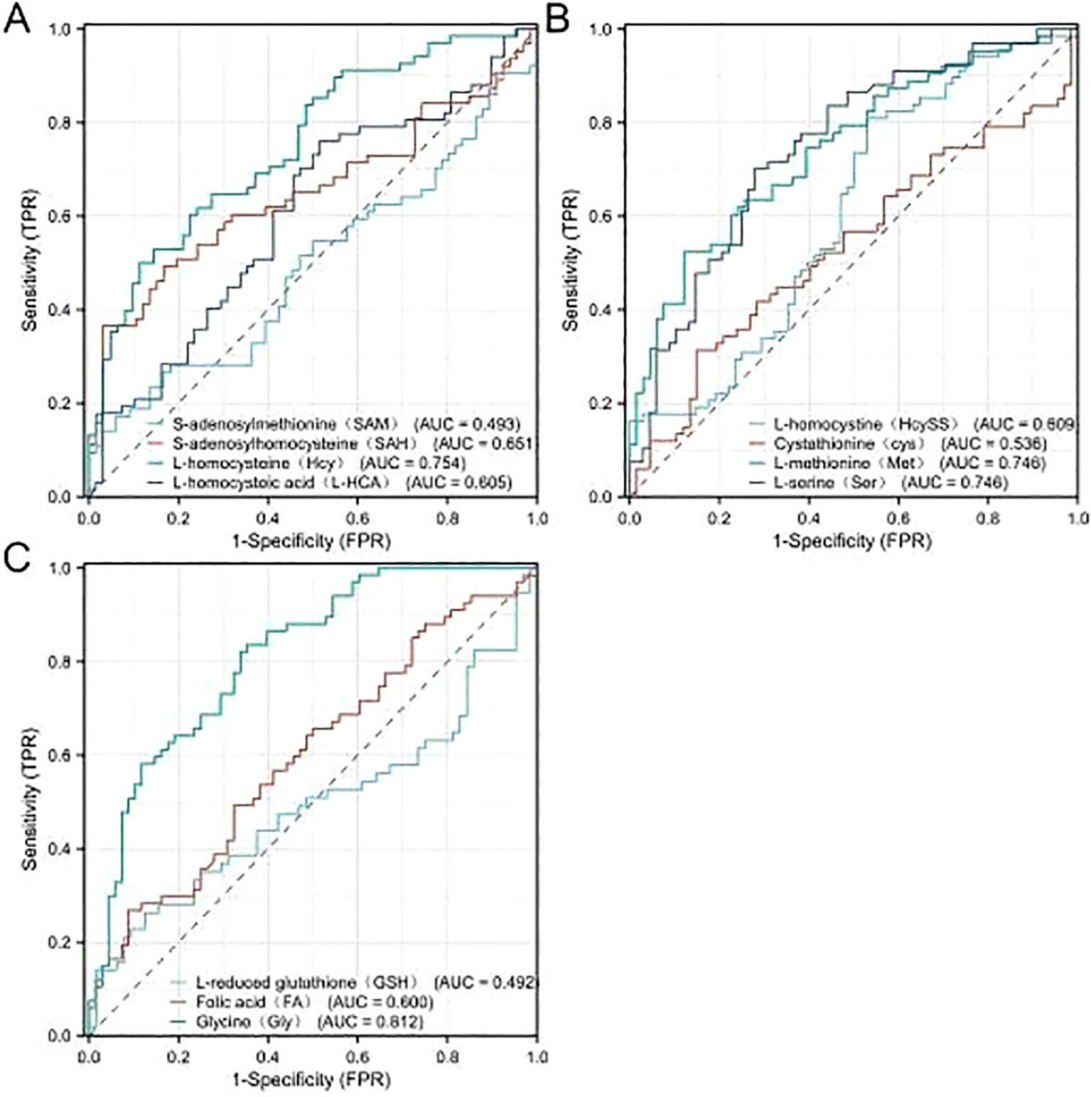
AUC values of various metabolites.

Serum concentrations of 11 one-carbon metabolism-related metabolites were quantified. The PDAC+NOD group showed reduced levels of multiple metabolites, including L-homocysteine, L-homocystine, L-methionine, L-serine, L-reduced glutathione, and glycine (**Supplementary Table 8**). These findings are consistent with the observed upregulation of key one-carbon metabolic enzymes (PHGDH, PSAT1, GLDC, SHMT1/2) in PDAC tumors from TCGA-PAAD data (**Supplementary Figure 5**), suggesting increased tumor utilization of circulating one-carbon metabolites. ROC analysis confirmed the discriminative capacity of these metabolic biomarkers, with individual AUC values detailed in **Figure 4**.

### 3.5 Machine learning model performance

Following feature selection, an optimal panel of five one-carbon metabolism metabolites was identified: glycine, L-serine, L-methionine, L-homocysteine, and L-homocystine (**Supplementary Figure 6A**). Among the four machine learning algorithms evaluated, the Gradient Boosting model demonstrated superior overall performance in distinguishing PDAC+NOD from NOD patients (**Table 1**). In a robust 5-fold cross-validation, this model achieved an area under the curve (AUC) of 0.853 (95% CI: 0.786-0.920), with 75.0% accuracy, 70.6% sensitivity, and 79.4% specificity. The detailed performance metrics for all evaluated models, including Random Forest, Support Vector Machine, and Logistic Regression, are presented in **Table 1**.

**Table 1:** Performance comparison of machine learning models using top 5 one-carbon metabolites.

Model	Accuracy	Precision	Recall (sensitivity)	Specificity	F1-score	AUC (Mean ± SD)	95% CI
Logistic Regression	0.765	0.772	0.799	0.731	0.765	0.848 ± 0.035	0.813-0.883
Random Forest	0.735	0.744	0.741	0.729	0.729	0.829 ± 0.063	0.766-0.892
Gradient Boosting	0.750	0.779	0.706	0.794	0.736	0.853 ± 0.067	0.786-0.920
SVM (RBF kernel)	0.750	0.730	0.813	0.687	0.759	0.829 ± 0.051	0.778-0.880

Values represent mean performance across 5-fold cross-validation. AUC, area under the receiver operating characteristic curve; CI, confidence interval; SD, standard deviation.

To assess the incremental diagnostic value of the biomarker panel, the 5-metabolite model was compared with the baseline model using only glycine. The 5-metabolite model significantly outperformed the glycine-only model (AUC 0.805, *p* < 0.001), as determined by DeLong’s test (**Supplementary Figure 6B**). The incremental value was further confirmed by a Net Reclassification Improvement (NRI) of 35.0% and an Integrated Discrimination Improvement (IDI) of 0.18 when compared to the glycine-only model, indicating a clinically meaningful improvement in risk stratification (**Table 2**).

**Table 2:** Baseline model comparison and incremental value metrics.

Model	Features	AUC	95% CI	Sensitivity	Specificity	Accuracy	NRI vs. model 3	IDI vs. model 3	*P*-value
Model 1	Glycine only	0.805	0.738-0.872	73.5%	76.5%	75.0%	35.0%	0.18	<0.001
Model 2	Age, BMI, DM duration	0.687	0.612-0.762	61.8%	67.6%	64.7%	48.5%	0.25	<0.001
Model 3	Top 5 metabolites (GB)	0.853	0.786-0.920	70.6%	79.4%	75.0%	—	—	—

DM, diabetes mellitus; GB, Gradient Boosting; NRI, Net Reclassification Improvement; IDI, Integrated Discrimination Improvement. P-values from DeLong test comparing Model 1 or 2 vs. Model 3.

The final model exhibited good calibration, with a Brier score of 0.185 and close alignment between predicted probabilities and observed frequencies on the calibration curve (**Supplementary Figure 6C**). Decision curve analysis showed a positive net benefit across a wide range of clinically relevant threshold probabilities (0.05 to 0.50), suggesting the model’s utility in guiding clinical decisions (**Supplementary Figure 6D**). When contextualized to real-world screening scenarios with PDAC prevalence rates of 0.5% and 1.0%, the model yielded an exceptionally high negative predictive value (NPV) of 99.8% and 99.6%, respectively. The corresponding positive predictive values (PPV) were 1.7% and 3.3%, underscoring the necessity of a two-step screening approach where this biomarker panel is used as a first-line test to rule out disease and select high-risk individuals for confirmatory imaging (**Table 3**).

**Table 3:** Positive and negative predictive values at real-world PDAC prevalence levels.

PDAC prevalence in NOD population	Sensitivity	Specificity	PPV	NPV	Number needed to screen*
0.5%	70.6%	79.4%	1.7%	99.8%	59
1.0%	70.6%	79.4%	3.3%	99.6%	30
5.0% (reference)	70.6%	79.4%	14.6%	98.3%	7

Number needed to screen*: Number of patients who need to undergo metabolite testing to detect one PDAC case, assuming all screen-positive individuals receive confirmatory imaging. PPV, positive predictive value; NPV, negative predictive value.

Sensitivity analyses confirmed the model’s robustness across different normalization methods and demographic subgroups. Importantly, stage-stratified performance evaluation demonstrated strong diagnostic capability for early-stage PDAC (Stage I-II: AUC = 0.841, 95% CI: 0.761-0.921), which holds particular clinical significance as these patients would benefit most from early intervention. The model showed enhanced performance in advanced-stage disease (Stage III-IV: AUC = 0.879, 95% CI: 0.782-0.976), likely reflecting more pronounced metabolic alterations (**Supplementary Table 9**). Furthermore, exploratory analysis revealed markedly improved performance in patients with elevated glycine/serine ratio (n=68), achieving an AUC of 0.876 (95% CI: 0.792-0.941) with 83.6% accuracy, 82.4% sensitivity, and 84.8% specificity (**Supplementary Figure 7**). This suggests the glycine/serine ratio may serve as a valuable stratification tool to identify patients who would derive maximum benefit from this diagnostic approach.

## 4 Discussion

PDAC remains one of the most aggressive malignancies, with a steadily rising global incidence and a five-year survival rate below 10% (1). Most patients are diagnosed at an advanced stage due to nonspecific early symptoms (1), highlighting the critical need for early detection strategies. Evidence suggests that diagnosing PDAC at resectable stages (T1N0M0) can increase five-year survival from 8% to 44% (24, 25), making early interception a key modifiable prognostic factor.

Our findings are consistent with and extend the growing body of metabolomic literature on PDAC. A recent multicenter study that integrated tissue and serum metabolomics also identified disruptions in one-carbon metabolism, including alterations in glycine and serine pathways, as key discriminants of PDAC, underscoring the fundamental role of these metabolic processes in pancreatic tumorigenesis (26). Furthermore, an untargeted metabolomics characterization of resectable PDAC confirmed significant metabolic reprogramming in early-stage disease, highlighting the potential of metabolomics to identify biomarkers for early detection (27). While these studies substantiate the broader relevance of the metabolic pathways we identified, our work specifically elucidates their diagnostic utility within the high-risk NOD cohort, a population with an urgent need for effective screening strategies.

The observed metabolic alterations highlight several key pathways implicated in PDAC pathogenesis within a diabetic context. Dysregulation of glutathione metabolism reflects disrupted redox homeostasis commonly seen in cancer (28–31), while perturbations in glycine/serine and tryptophan metabolism suggest increased nucleotide synthesis demand and immune microenvironment modulation, respectively (32, 33). Central to these changes is one-carbon metabolism, which integrates serine/glycine metabolism with folate and methionine cycles to support nucleotide synthesis and epigenetic regulation through S-adenosylmethionine production (34–40). The significant reduction in one-carbon related metabolites (glycine, L-serine, L-methionine, L-homocysteine, and L-homocystine) in PDAC+NOD patients aligns with increased tumor utilization of these substrates, offering both diagnostic insights and potential therapeutic targets for early detection in high-risk populations. In summary, the increased demand for one-carbon metabolism associated with tumor proliferation may lead to an elevated uptake of related amino acids from the bloodstream, potentially resulting in decreased circulating levels of these metabolites. This is consistent with our findings, which showed significant decreases in serum levels of glycine, L-serine, L-methionine, L-homocysteine, and L-homocystine in the PDAC with diabetes group.

The diagnostic performance of our 5-metabolite panel (AUC = 0.876, 95% CI: 0.792-0.941; sensitivity = 82.4%, specificity = 83.6%) demonstrates considerable promise for early PDAC detection in high-risk NOD patients (5, 29). While the current specificity warrants careful consideration for screening applications, it can be optimized through threshold adjustment. The panel’s high sensitivity renders it particularly suitable as an initial test within a sequential screening strategy. This approach becomes especially relevant given that CA19-9, while showing elevated expression in advanced disease, demonstrates limited sensitivity (50%-60%) for early-stage (I/II) PDAC, making it inadequate as a standalone screening tool. Although direct comparison with CA19–9 was constrained by cohort characteristics, our panel’s performance compares favorably with literature-reported CA19–9 accuracy while simultaneously providing valuable biological insights into PDAC metabolic dysregulation. The age restriction (>65 years) enhances internal validity while limiting generalizability; consequently, future validation in broader populations—including younger NOD patients and those with normal CA19–9 levels—remains essential to establish clinical utility and demonstrate incremental value over existing biomarkers (18, 21).

Several limitations should be considered when interpreting our findings. First, our study did not fully adjust for all potential clinical confounders, including medications and comorbidities, in the multivariable model due to sample size constraints. While subgroup analyses suggested consistent performance across these variables, future studies with larger cohorts should incorporate comprehensive adjustment for these factors to confirm the independence of the metabolic signature. Second, the limited sample size from a single institution constrains the generalizability of our findings, necessitating external validation to confirm the diagnostic performance of our metabolite panel. Third, our study lacks epidemiological data on the progression of NOD to PDAC in the enrolled population, which would have provided valuable context for risk stratification. Future multi-center studies with larger sample sizes and prospective validation are needed to confirm the clinical utility of the proposed biomarkers. Additionally, collecting longitudinal data on NOD populations will be essential for establishing more accurate PDAC risk prediction models.

In summary, this study highlights the potential of a serum metabolomic signature for early PDAC detection in high-risk NOD patients. However, limitations include the single-center design, modest sample size, and lack of external validation. Further validation in larger, multi-center cohorts and longitudinal studies is needed to establish clinical utility.

## Data Availability

The datasets presented in this study can be found in online repositories. The names of the repository/repositories and accession number(s) can be found in the article/**Supplementary Material.**
