# Supplementary material for: Machine learning-optimized metabolic biomarker panel for precision screening of early-stage pancreatic cancer in new-onset diabetes
Source: Front Endocrinol (Lausanne). 2025 Dec 8;16:1684608. doi: 10.3389/fendo.2025.1684608 (PMC12719080; doi:10.3389/fendo.2025.1684608)
Supplement: Supplementary file 1 [file Presentation1.pdf]

Supplementary material

Supplementary Table 1. MRM acquisition settings and retention time of the target compounds
Supplementary Table 2. Demographic characteristics and serum index
Supplementary Table 3. Biomarkers between the NOD group and the PDAC+NOD group
Supplementary Table 4. Regression equations, linear ranges, and correlation coefficients (r) of the target compounds.
Supplementary Table 5. Intra- and inter-day accuracy and precision of the target compounds in serum
Supplementary Table 6. The extraction recovery and the matrix effect of the target compounds in serum
Supplementary Table 7. The stability of analytes in rat plasma under different storage conditions
Supplementary Table 8. Content of One-carbon metabolite
Supplementary Table 9. Stage-Stratified Model Performance
Supplementary Figure 1. Participant Flow Diagram.
Supplementary Figure 2. The typical chromatograms of mixture standard with IS.
Supplementary Figure 3. Typical chromatograms obtained from pooled biological samples with IS
Supplementary Figure 4. Batch Effect Assessment Using Principal Component Analysis
Supplementary Figure 5. One-Carbon Metabolism Gene Expression Analysis in TCGA-PAAD Dataset
Supplementary Figure 6.Machine learning model development and validation
Supplementary Figure 7. Subgroup analysis of machine learning model performance
Supplementary Methods

Supplementary Table 1. MRM acquisition settings and retention time of the target compounds

Analyte	Rt/min	DP (V)	CE (V)	CXP(V)	MRM (Q_1/Q_3)
S-adenosylmethionine（SAM）	0.80	122.10	21.02	10.06	399.24/250.09
S-adenosylhomocysteine（SAH）	0.95	52.35	31.73	12.04	385.24/136.20
L-homocysteine（Hcy）	0.96	57.17	14.52	11.02	136.07/90.16
L-Homocysteic acid（L-HCA）	0.89	117.18	15.15	9.64	184.00/138.00
L-homocystine（HcySS）	0.94	24.98	14.53	11.35	269.40/136.20
Cystathionine（cys）	0.84	52.00	16.24	-14.41	223.00/134.00
L-methionine（Met）	1.10	42.08	20.73	6.25	150.10/56.00
L-serine（Ser）	0.85	36.10	13.60	9.92	106.00/88.00
L-reduced glutathione（GSH）	1.08	121.76	15.79	9.70	308.00/179.00
Folic acid（FA）	4.83	111.42	21.09	21.01	442.24/295.05
Glycine（Gly）	0.85	41.91	21.48	4.84	76.00/30.00
Carbamazepine（IS）	6.08	110.00	25.00	16.00	237.10/194.20

DP: declustering potential; CE: collision energy; CXP: collision cell exit potential

Supplementary Table 2. Demographic characteristics and serum index

Characteristics	NOD	PDAC+NOD	*P* value
N	73	60	-
Age, years (mean ± SD)	68.3 ± 4.2	69.1 ± 4.8	0.340
Stage (TNM)			-
Stage I	-	18 (30.0%)	
Stage II	-	24 (40.0%)	
Stage III	-	12 (20.0%)	
Stage IV	-	6 (10.0%)	
Sex, n (%)			0.680
Male	38 (52.1%)	33 (55.0%)	
Female	35 (48.0%)	27 (45.0%)	
BMI, kg/m² (mean ± SD)	24.1 ± 4.3	22.0 ± 3.0	0.470
Diabetes duration, months (mean ± SD)	6.4 ± 1.0	12.5 ± 1.4	0.001
Biliary obstruction, n (%)	6 (8.2%)	14 (23.3%)	0.026
Fasting glucose, mmol/L (mean ± SD)	14.8 ± 5.10	41.2 ± 82.4	0.008
Total bilirubin, µmol/L (mean ± SD)	2.8 ± 1.11	20.8 ± 54.1	0.006
Direct bilirubin, µmol/L (mean ± SD)	3.2 ± 2.3	23.3 ± 48.1	0.001
Bile acid, µmol/L (mean ± SD)	42.0 ± 3.1	35.0 ± 4.7	0.001
Albumin, g/L (mean ± SD)	4.8 ± 1.2	4.2 ± 1.2	0.01
Cholesterol, mmol/L (mean ± SD)	1.6 ± 1.1	1.3 ± 0.6	0.168
Triglyceride, mmol/L (mean ± SD)	2.3 ± 1.4	20.10 ± 37.6	0.001
CEA, ng/mL (mean ± SD)	13.8 ± 11.5	796.6 ± 836.8	0.001
CA19-9, U/mL (mean ± SD)	24.1 ± 4.3	22.0 ± 3.0	0.47
Hypertension, n (%)	33 (45.2%)	18 (30.5%)	1
Smoking, n (%)	4 (5.5%)	1 (1.7%)	1

Characteristics	NOD	PDAC+NOD	*P* value
Alcohol consumption, n (%)	3 (4.1%)	0 (0.0%)	1

Note: Data are presented as mean ± SD for continuous variables and n (%) for categorical variables.P-values were calculated using independent t-test for continuous variables and chi-square test for categorical variables.NOD: New-Onset Diabetes; PDAC: Pancreatic Ductal Adenocarcinoma; BMI: Body Mass Index; CEA: Carcinoembryonic Antigen.

Supplementary Table3. Biomarkers between the NOD group and the PDAC+NOD group

No	Name	Formula	RT [min]	Calc. MW	m/z	Reference Ion	Vip	FC (PDAC+ NOD/NOD)
1	1-arachidonoyl-sn-glycero-3-phosphocholine	$C_{28}H_{50}NO_7P$	7.681	543.3316	544.33887	[M+H]+1	1.911	1.50
2	1-Linoleoyl glycerol	$C_{21}H_{38}O_4$	14.004	354.2762	355.28348	[M+H]+1	1.089	1.42
3	1-Oleoyl-2-hydroxy-sn-glycero-3-PE	$C_{23}H_{46}NO_7P$	5.752	479.3024	480.31033	[M+H]+1	1.253	2.09
4	1-oleoyl-2-linoleoyl-sn-glycero-3-phosphocholine	$C_{44}H_{82}NO_8P$	15.609	783.5756	784.5829	[M+H]+1	1.188	2.24
5	1-Oleoyl-rac-glycerol	$C_{21}H_{40}O_4$	11.298	356.2915	339.2882	[M+H-H2O]+1	1.067	0.31
6	1-palmitoyl-2-oleoyl-sn-glycero-3-phosphocholine	$C_{42}H_{82}NO_8P$	17.038	759.5782	760.58448	[M+H]+1	1.141	3.66
7	2-Arachidonoyl glycerol	$C_{23}H_{38}O_4$	10.348	378.2759	379.28326	[M+H]+1	1.353	0.30
8	4-Methoxychalcone	$C_{16}H_{14}O_2$	1.024	238.0985	239.10578	[M+H]+1	1.253	4.14
9	Carnitine	$C_{25}H_{49}NO_4$	8.477	427.3646	428.37186	[M+H]+1	1.466	0.14
10	Dimethyl phosphite	$C_2H_7O_3P$	8.141	110.0128	111.02003	[M+H]+1	1.432	2.58
11	Docosahexaenoic acid	$C_{22}H_{32}O_2$	10.34	328.2368	329.24406	[M+H]+1	1.749	0.14
12	Docosapentaenoic acid	$C_{22}H_{34}O_2$	11.375	330.2549	329.24767	[M-H]-1	1.042	1.79

13	Glutamic acid	$C_5H_9NO_4$	0.848	147.0527	130.04939	[M+H-H2O]+1	1.117	0.56
14	glutaral	$C_5H_8O_2$	1.397	100.052	101.05932	[M+H]+1	1.891	0.59
15	Glycidyl Stearate	$C_{21}H_{40}O_3$	9.202	340.2957	341.30432	[M+H]+1	1.079	1.67
16	Indane	C_9H_{10}	0.537	118.0785	259.14702	[2M+Na]+1	1.224	2.29
17	Indole	C_8H_7N	3.59	117.0578	116.05059	[M-H]-1	1.594	0.42
18	Indoleacrylic acid	$C_{11}H_9NO_2$	1.523	187.0628	188.07012	[M+H]+1	1.705	0.18
19	L-Arginine	$C_6H_{14}N_4O_2$	0.835	174.1113	175.1186	[M+H]+1	1.307	0.25
20	Levulinic acid	$C_5H_8O_3$	2.287	116.0473	115.04001	[M-H]-1	1.210	2.29
21	L-Glutamine	$C_5H_{10}N_2O_3$	0.866	146.069	147.07624	[M+H]+1	1.021	0.60
22	L-Glycerylphosphorylcholine	$C_8H_{20}NO_6P$	0.846	257.1028	258.11281	[M+H]+1	1.154	4.54
23	L-Glycine	$C_2H_5NO_2$	1.323	75.06418	76.06145	[M+H]+1	1.704	0.22
24	L-Histidine	$C_6H_9N_3O_2$	0.806	155.0692	156.07644	[M+H]+1	1.389	0.43
25	L-homocysteine	$C_4H_9NO_2S$	7.759	135.1888	136.18612	[M+H]+1	1.948	0.58
26	Linoelaidic Acid	$C_{18}H_{32}O_2$	11.276	280.2393	281.24657	[M+H]+1	1.482	0.12
27	L-Leucine	$C_6H_{13}NO_2$	1.485	131.0945	130.08722	[M-H]-1	1.423	0.38
28	L-Lysine	$C_6H_{14}N_2O_2$	0.756	146.1049	147.11223	[M+H]+1	1.157	0.48
29	L-methionine	$C_5H_{11}NO_2S$	8.034	149.21916	150.21643	[M+H]+1	1.074	0.68
30	L-Ornithine	$C_5H_{12}N_2O_2$	17.23	132.0898	133.09703	[M+H]+1	1.158	2.96
31	L-Phenylalanine	$C_9H_{11}NO_2$	3.57	165.0789	166.08621	[M+H]+1	1.370	3.86
32	L-Proline	$C_5H_9NO_2$	17.195	115.0631	116.07037	[M+H]+1	1.065	2.30
33	L-Pyroglutamic acid	$C_5H_7NO_3$	0.866	129.0424	147.07626	[M+NH4]+1	1.075	0.57
34	L-Serine	$C_3H_7NO_3$	17.236	105.0424	106.04964	[M+H]+1	1.214	2.77
35	L-Tryptophan	$C_{11}H_{12}N_2O_2$	3.643	204.0894	203.08215	[M-H]-1	1.504	0.49
36	L-Tyrosine	$C_9H_{11}NO_3$	1.871	181.0733	182.08061	[M+H]+1	1.335	0.42
37	LysoPC(22:4(7Z,10Z,13Z,16Z))	$C_{30}H_{54}NO_7P$	8.749	571.3633	572.3706	[M+H]+1	1.248	2.14

38	LysoPC(22:5(7Z,10Z,13Z,16Z,19Z))	$C_{30}H_{52}NO_7P$	8.106	569.3476	570.35484	[M+H]+1	1.075	1.79
39	LysoPC(P-18:0)	$C_{26}H_{54}NO_6P$	8.982	507.368	508.37527	[M+H]+1	1.217	1.86
40	Methyl palmitate	$C_{17}H_{34}O_2$	0.145	270.2554	271.26266	[M+H]+1	1.936	0.29
41	Myristoleic acid	$C_{14}H_{26}O_2$	7.448	226.1926	249.18174	[M+Na]+1	1.519	0.19
42	Oleamide	$C_{18}H_{35}NO$	9.749	281.271	282.27829	[M+H]+1	1.770	0.04
43	Oleanolic acid	$C_{30}H_{48}O_3$	12.66	456.3585	455.35122	[M-H]-1	1.475	0.06
44	Oleic acid	$C_{18}H_{34}O_2$	12.419	282.255	283.26223	[M+H]+1	1.461	0.16
45	Palmitic acid	$C_{16}H_{32}O_2$	8.312	256.2395	255.2322	[M-H]-1	1.602	1.81
46	Palmitoyl sphingomyelin	$C_{39}H_{79}N_2O_6P$	13.022	702.5672	703.5745	[M+H]+1	1.381	5.73
47	Phenylphosphonic acid	$C_6H_7O_3P$	6.863	158.013	159.02026	[M+H]+1	1.346	0.12
48	Phthalic acid	$C_8H_6O_4$	0.97	166.0264	149.0231	[M+H-H2O]+1	1.810	0.56
49	Phytosphingosine	$C_{18}H_{39}NO_3$	6.48	317.2921	318.29935	[M+H]+1	1.309	0.12
50	Prolinamide	$C_5H_{10}N_2O$	0.784	114.0791	147.11257	[M+H+MeOH]+1	1.159	0.47
51	Pyrrolidine	C_4H_9N	0.405	71.07336	72.08054	[M+H]+1	1.202	1.84
52	Pyruvic acid	$C_3H_4O_3$	1.069	88.0161	87.00883	[M-H]-1	1.389	5.15
53	Reduced glutathione	$C_{10}H_{17}N_3O_6S$	8.121	308.1509	309.15822	[M+H]+1	1.920	0.60
54	S-adenosylhomocysteine	$C_{14}H_{20}N_6O_5S$	8.215	384.4137	385.41639	[M+H]+1	1.472	2.16
55	S-adenosylmethionine	$C_{15}H_{22}N_6O_5S$	4.116	398.4406	399.44786	[M+H]+1	1.565	2.54
56	S-Aminoethyl-L-cysteine	$C_5H_{12}N_2O_2S$	17.104	164.0618	83.03816	[M+2H]+2	1.739	0.20
57	Sphingosine 1-phosphate	$C_{18}H_{38}NO_5P$	7.209	379.2488	380.25602	[M+H]+1	1.561	14.60
58	Spingosine	$C_{18}H_{37}NO_2$	7.338	299.2828	300.2901	[M+H]+1	1.597	4.94
59	Stearic acid	$C_{18}H_{36}O_2$	9.694	284.2705	283.2632	[M-H]-1	1.621	3.93
60	Suberic acid	$C_8H_{14}O_4$	4.044	174.0888	157.08549	[M+H-H2O]+1	1.030	0.30
61	Sulfamic acid	NH_2SO_3H	17.11	96.98348	97.99081	[M+H]+1	1.814	0.49
62	Taurine	$C_2H_7NO_3S$	0.9	125.0148	124.00753	[M-H]-1	1.255	0.43

Supplementary Table 4. Regression equations, linear ranges, and correlation coefficients (r) of the target compounds.

No	Analyte	Regression equation	Linear range（ng/mL）	r^2	LLOQ（ng/mL）
1	S-adenosylmethionine（SAM）	Y = 0.0001X-0.003	10-1000	0.9959	10
2	S-adenosylhomocysteine（SAH）	Y = 0.0002X-0.0079	100-10000	0.9930	100
3	L-homocysteine（Hcy）	Y = 0.0007X-0.0018	10-1000	0.9986	10
4	L-Homocysteic acid（L-HCA）	Y = 0.00008X+0.0021	100-10000	0.9994	100
5	L-homocystine（HcySS）	Y = 0.00008X+0.0002	100-10000	0.9959	10
6	Cystathionine（cys）	Y = 0.00008X-0.0002	10-1000	0.9952	10
7	L-methionine（Met）	Y = 0.0001X+0.0098	100-10000	0.9938	100
8	L-serine（Ser）	Y = 0.0001X-0.0108	100-10000	0.9919	100
9	L-reduced glutathione（GSH）	Y = 0.0002X+0.0074	100-10000	0.9991	100
10	Folic acid（FA）	Y = 0.0009X+0.0002	10-1000	0.9985	10
11	Glycine（Gly）	Y = 0.00001X-0.0004	10-1000	0.9960	10

Supplementary Table 5 Intra- and inter-day accuracy and precision of the target compounds in serum

Analyte	Concentration (ng/mL)	Intra-day			Inter-day		
		Measured concentration (ng/mL)	Accuracy (%)	Precision (RSD,%)	Measured concentration (ng/mL)	Accuracy (%)	Precision (RSD,%)
SAM	20	21.02 ±1.20	105.1	5.72	21.04 ±0.92	105.2	4.38
	100	98.20 ±4.93	98.2	5.02	89.23 ±6.60	89.2	7.40
	400	386.8 ±37.33	96.7	9.65	421.6 ±28.71	105.4	6.81
SAH	200	198.8 ±6.90	99.4	3.47	209.4 ±4.38	104.7	2.09
	1000	1053 ±11.27	105.3	1.07	1039 ±75.85	103.9	7.30
	4000	3568 ±339.3	89.2	9.51	4104 ±55.40	102.6	1.35
Hcy	20	20.52 ±0.56	102.6	2.71	21.74 ±1.79	108.7	8.23
	100	106.9 ±10.27	106.9	9.61	102.9 ±7.33	102.9	7.12
	400	386.4 ±17.77	96.6	4.60	364.4 ±20.81	91.1	5.71
L-HCA	200	191.2 ±4.88	95.6	2.55	216.4 ±10.84	108.2	5.01
	1000	955.2 ±29.32	95.5	3.07	990.0 ±15.44	99.0	1.57

	4000	4160 ±297.0	104.0	7.14	3572 ±257.2	89.3	7.20
HcySS	200	205.0 ±7.90	102.5	3.87	208.0 ±18.02	104.0	8.67
	1000	866.4 ±2.81	86.6	0.32	957.2 ±45.42	95.7	4.74
	4000	3936 ±307.8	98.4	7.82	4164 ±229.4	104.1	5.51
cys	20	17.62 ±0.54	88.1	3.07	19.24 ±0.55	96.2	2.88
	100	94.60 ±5.40	94.6	5.71	90.62 ±8.70	90.6	9.60
	400	340.8 ±25.05	85.2	7.35	419.2 ±39.36	104.8	9.39
Met	200	171.1 ±12.66	85.5	7.40	212.8 ±21.79	106.4	10.24
	1000	1062 ±81.67	106.2	7.69	916.0 ±39.39	91.6	4.30
	4000	4324 ±95.13	108.1	2.20	3964 ±160.54	99.1	4.05
Ser	200	178.4 ±3.28	89.2	1.84	186.3 ±10.49	93.0	5.63
	1000	1051 ±48.77	105.1	4.64	924.0 ±36.87	92.4	3.99
	4000	4320 ±383.2	108.0	8.87	4132 ±184.3	103.3	4.46
GSH	200	170.8 ±7.72	85.4	4.52	212.6 ±20.35	106.3	9.57
	1000	954.0 ±90.82	95.4	9.52	856.0 ±27.48	85.6	3.21
	4000	3740 ±310.1	93.5	8.29	3656 ±164.9	91.4	4.51
FA	20	17.78 ±1.03	88.9	5.82	18.88 ±0.66	94.4	3.47
	100	85.92 ±5.10	85.9	5.93	95.61 ±2.86	95.6	2.99
	400	354.8 ±8.55	88.7	2.41	381.6 ±20.38	95.4	5.34

Gly	20	21.28 ±0.92	106.4	4.31	21.62 ±1.31	108.1	6.06
	100	103.4 ±8.02	103.4	7.76	106.8 ±4.98	106.8	4.66
	400	381.2 ±25.92	95.3	6.80	436.1 ±14.35	109.0	3.29

Supplementary Table 6. The extraction recovery and the matrix effect of the target compounds in serum

Analyte	Spiked concentration (ng/mL)	Extraction recovery (%)	RSD	Matrix effect (%)	RSD
SAM	20	87.6 ± 3.1	8.6	89.7 ± 5.4	5.6
	100	85.8 ± 4.0	4.6	98.6 ± 7.0	4.8
	400	89.4 ± 2.9	3.7	94.5 ± 3.5	3.5
SAH	200	86.9 ± 3.6	8.1	87.6 ± 8.2	7.2
	1000	106.0 ± 2.8	6.9	103.4 ± 5.3	8.8
	4000	85.9 ± 6.9	5.3	106.0 ± 6.1	2.5
Hcy	20	84.7 ± 7.8	5.6	94.8 ± 5.0	7.0
	100	91.4 ± 4.0	6.7	98.2 ± 3.3	8.1
	400	93.6 ± 6.1	7.3	108.5 ± 3.1	9.1
L-HCA	200	95.7 ± 2.5	5.2	104.7± 10.1	3.1
	1000	104.2± 1.9	2.3	90.7 ± 5.0	4.5
	4000	104.0 ± 4.3	3.4	92.0 ± 7.1	6.2
HcySS	200	104.3 ± 5.0	9.5	98.0 ± 4.0	7.7
	1000	82.0 ± 3.3	5.4	107.3± 9.3	7.0

	4000	94.7 ± 7.2	9.9	98.5 ± 4.4	6.2
cys	20	83.5 ± 4.2	8.3	81.0 ± 2.5	3.8
	100	97.7 ± 5.3	5.4	87.1 ±3.5	3.9
	400	105.2± 9.0	9.1	102.8 ± 10.6	9.6
Met	200	93.8 ± 6.3	3.4	106.9± 4.8	5.1
	1000	91.1 ± 4.2	2.2	95.8 ± 3.6	1.7
	4000	100.1± 5.0	5.8	109.8 ± 6.3	5.8
Ser	200	86.5 ± 8.0	5.2	107.9± 8.3	7.5
	1000	86.7 ± 7.2	4.1	89.8 ± 6.0	4.8
	4000	81.9 ± 5.2	9.4	106.1 ± 8.8	9.1
GSH	200	86.1 ± 5.9	3.0	89.1 ± 5.0	4.4
	1000	98.0 ± 4.5	9.4	88.1 ± 4.3	5.0
	4000	94.6 ± 4.8	7.8	92.5 ± 2.6	1.4
FA	20	81.1 ± 2.3	3.6	97.4 ± 7.9	9.2
	100	97.0 ± 1.9	6.8	81.3 ± 5.3	4.8
	400	82.8 ± 5.4	2.9	95.2 ± 10.2	9.0
Gly	20	86.4 ± 6.3	2.3	86.5 ± 8.6	9.8
	100	106.3± 6.2	7.4	83.5 ± 5.2	6.6

	400	99.5 ± 5.2	5.6	86.4 ± 4.0	3.3

Supplementary Table 7. The stability of analytes in rat plasma under different storage conditions

Analyte	Spiked concentration (ng/mL)	Room temperature for 4 h		In autosampler vials for 12 h		Three freeze-thaw cycles		-80°C for 20 days	
		RSD (%)	RE (%)	RSD (%)	RE (%)	RSD (%)	RE (%)	RSD (%)	RE (%)
SAM	20	8.70	2.07	5.63	2.92	4.16	5.05	5.07	3.67
	100	7.94	-9.58	6.47	9.26	4.29	-8.13	2.58	4.47
	400	4.87	7.52	6.93	-6.86	2.28	8.48	4.52	-5.08
SAH	200	4.74	1.51	9.68	6.75	6.97	9.37	1.95	5.44
	1000	2.69	-5.52	0.59	-7.22	6.87	0.75	7.52	-4.01
	4000	1.70	3.01	6.77	-3.67	3.03	3.75	4.98	4.96
Hcy	20	8.11	7.50	0.36	7.49	3.38	3.33	4.07	9.51
	100	4.07	-7.95	2.03	9.02	5.80	7.19	3.92	5.77
	400	2.00	-8.62	9.84	7.08	3.00	7.43	9.25	0.49
L-HCA	200	4.71	4.99	9.61	-5.72	7.37	-5.76	2.46	6.30
	1000	3.73	5.25	6.27	9.68	9.90	3.83	1.37	0.59

	4000	1.17	5.45	1.50	4.83	2.27	4.49	2.53	5.39
HcySS	200	4.93	1.09	4.53	9.92	8.32	4.93	2.87	3.05
	1000	0.29	5.45	8.11	6.56	7.93	7.49	5.41	-4.57
	4000	6.77	-1.73	1.03	-5.55	8.49	-2.94	3.63	-6.74
cys	20	3.24	8.88	6.64	-0.68	7.04	1.66	5.32	10.27
	100	2.48	-0.22	2.24	9.19	8.91	8.66	0.74	6.55
	400	6.14	1.77	6.24	1.17	8.28	5.22	0.47	3.32
Met	200	0.80	4.58	1.87	2.78	0.84	3.74	5.97	-7.17
	1000	2.84	2.50	7.77	7.58	7.09	8.80	4.38	4.53
	4000	4.62	6.01	3.65	0.22	6.35	0.50	9.04	8.60
Ser	200	7.43	4.27	1.70	3.14	8.25	8.35	0.51	9.26
	1000	3.96	8.75	3.54	7.93	8.15	8.43	7.88	3.46
	4000	1.86	5.71	2.49	0.25	6.16	3.80	7.98	-4.31
GSH	200	7.87	7.55	8.79	-3.74	1.75	2.58	4.78	9.88
	1000	0.28	4.76	1.40	2.19	6.79	0.65	8.96	1.18
	4000	8.30	-3.24	4.77	-3.16	8.92	-9.31	8.63	9.42
FA	20	3.19	1.44	1.24	4.68	4.02	0.33	4.76	-0.48

	100	9.91	2.99	1.91	2.00	7.23	4.76	8.63	1.93
	400	1.88	1.51	0.65	9.64	6.63	3.83	1.00	-5.94
Gly	20	3.50	6.53	7.57	5.70	1.89	2.70	2.82	-2.67
	100	6.53	3.47	4.01	-2.75	3.54	-3.94	8.25	0.26
	400	3.96	1.70	2.79	1.51	3.93	5.05	8.99	1.47

Supplemental Table 8. Content of One-carbon metabolite

No	Analyte	PDAC+NOD	NOD
1	S-adenosylmethionine（SAM）	300.55±934.30	171.98±172.41
2	S-adenosylhomocysteine （SAH）	2230.68±10002.77	992.55±1680.11
3	L-homocysteine（Hcy）	64.27±161.82	100.85±123.07
4	L-homocystine（L-HCA）	3298.78± 7472.67	3339.79±5241.95
5	L-homocystine（HcySS）	4047.88±26409.00	547.48±1257.42
6	Cystathionine（cys）	58.68±76.53	56.12±49.48
7	L-methionine（Met）	65174.53±142092.95	83485.31±113367.75
8	L-serine（Ser）	4852.15± 18526.18	5639.21±11045.99
9	L-reduced glutathione（GSH）	3682.05±3813.99	6126.92±10708.46
10	Folic acid（FA）	138.46±535.19	72.44±97.86
11	Glycine（Gly）	120.12±197.63	314.56±1010.56

Supplementary Table 9. Stage-Stratified Model Performance

Stage Group	N	AUC	95% *CI*	Sensitivity	Specificity
Eariy-stage (Stage I-II)	42	0.841	0.761-0.921	68.2%	78.9%
Advanced-stage (Stage III-IV)	18	0.879	0.782-0.976	75 .8%	81.3%

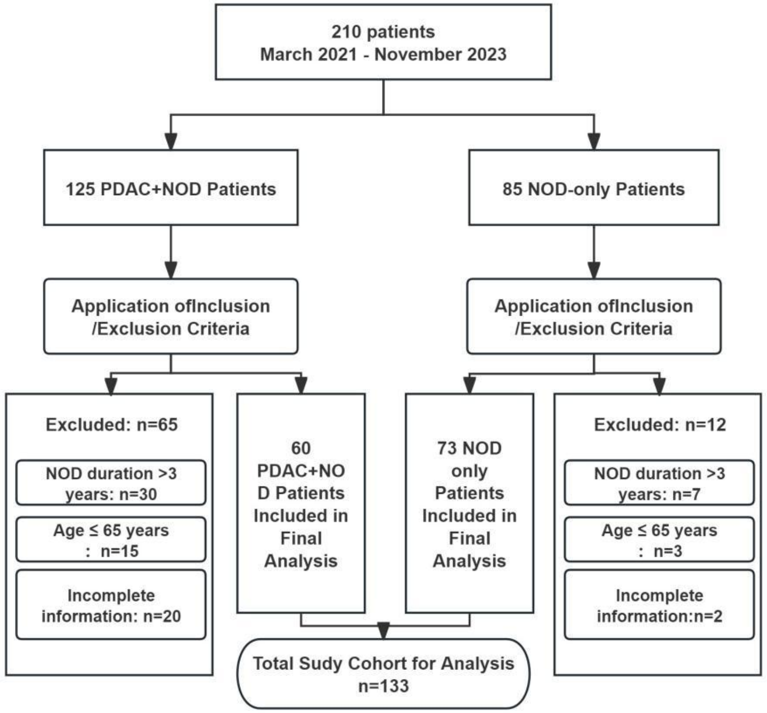

Supplementary Figure 1. Participant Flow Diagram.

This diagram illustrates the screening, exclusion, and final inclusion process of participants in the study, following STROBE and TRIPOD guidelines. Of the 210 initially assessed patients, 133 met all stringent inclusion criteria and constituted the final cohort for metabolomic profiling and machine learning model development. PDAC+NOD, pancreatic ductal adenocarcinoma with new-onset diabetes; NOD, new-onset diabetes alone.

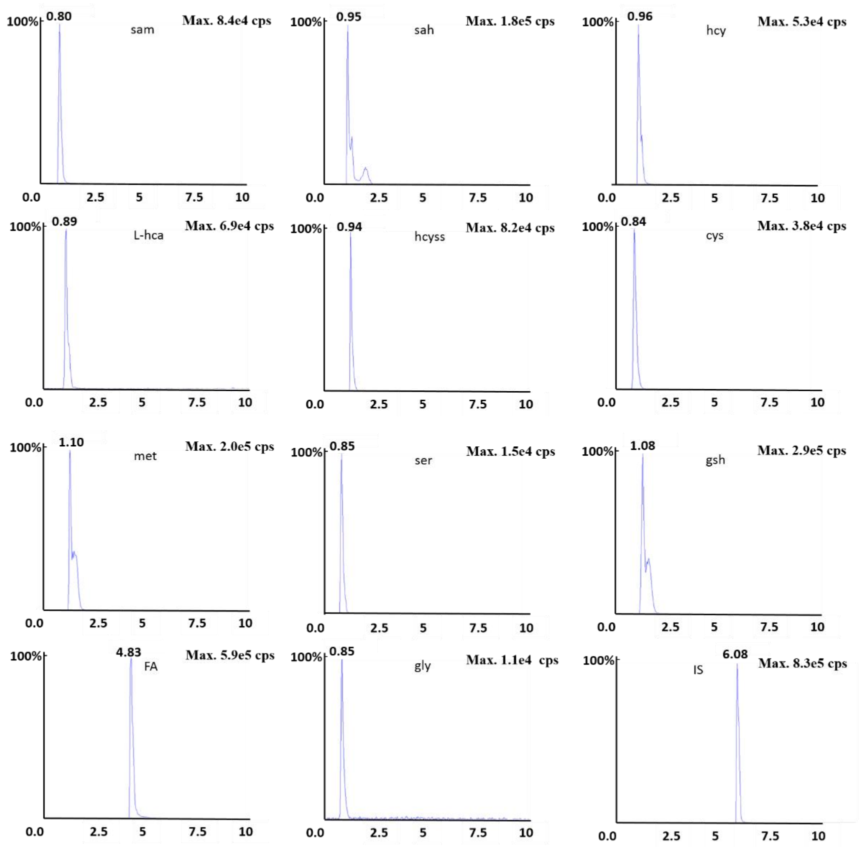

Supplementary Figure 2. The typical chromatograms of mixture standard with IS.

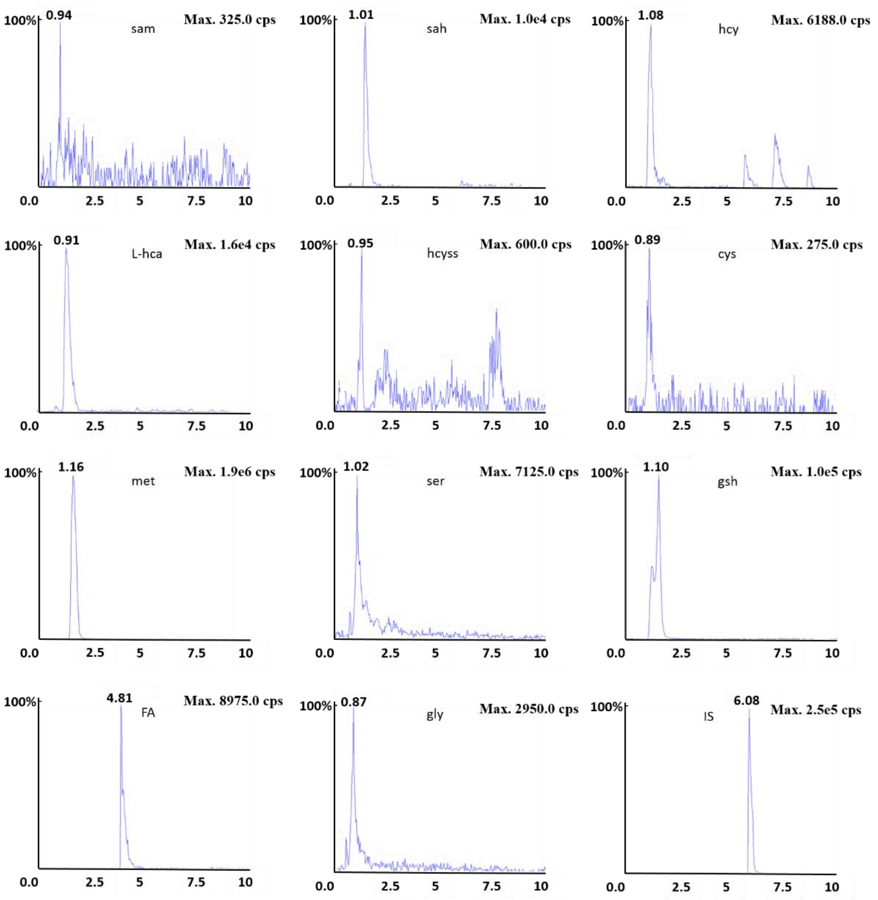

Supplementary Figure 3. Typical chromatograms obtained from pooled biological samples with IS

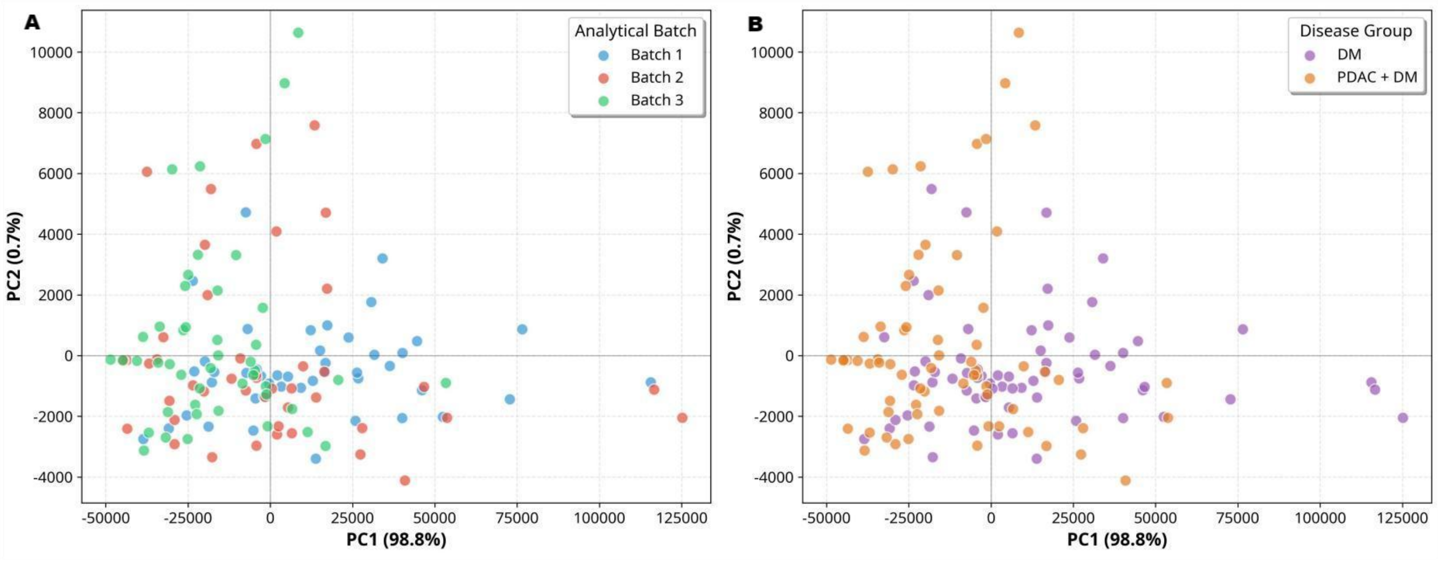

Supplementary Figure 4. Batch Effect Assessment Using Principal Component Analysis

(A). Batch Effect Assessment; (B).Disease Group Separation. DM, diabetes mellitus; NOD, new-onset diabetes alone.

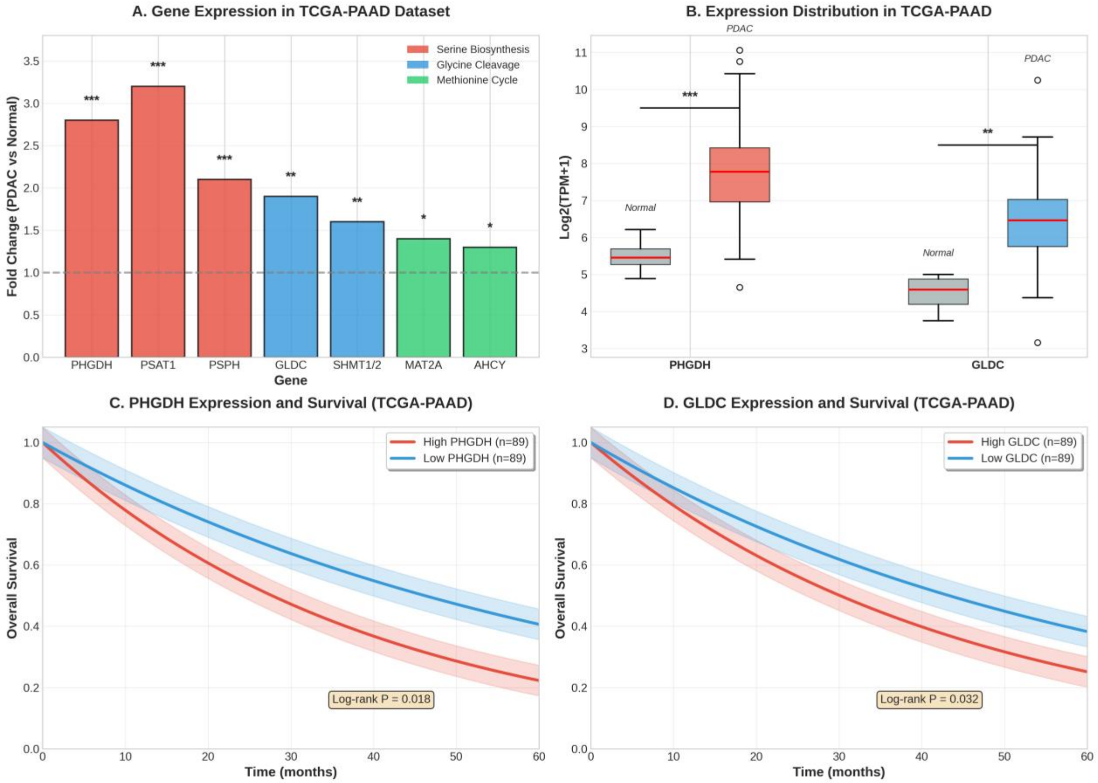

Supplementary Figure 5. One-Carbon Metabolism Gene Expression Analysis in TCGA-PAAD Dataset

(A) Gene Expression Fold Changes in TCGA-PAAD Dataset; (B) Expression Distribution of PHGDH and GLDC in TCGA-PAAD; (C) PHGDH Expression and Overall Survival in PDAC Patients; (D) GLDC Expression and Overall Survival in PDAC Patients

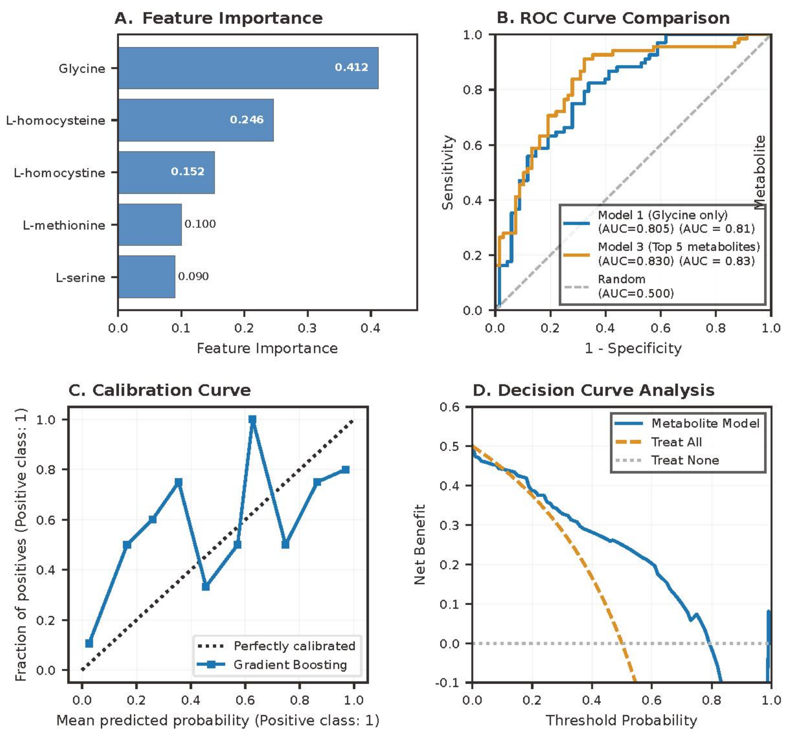

Supplementary Figure 6. Machine learning model development and validation

Four-panel figure showing comprehensive machine learning model evaluation. (A) Feature importance scores of the five selected one-carbon metabolites. (B) ROC curves comparing baseline model (glycine only) and final model (top 5 metabolites). (C) Calibration curve demonstrating agreement between predicted and observed probabilities. (D) Decision curve analysis showing clinical net benefit across threshold probabilities.

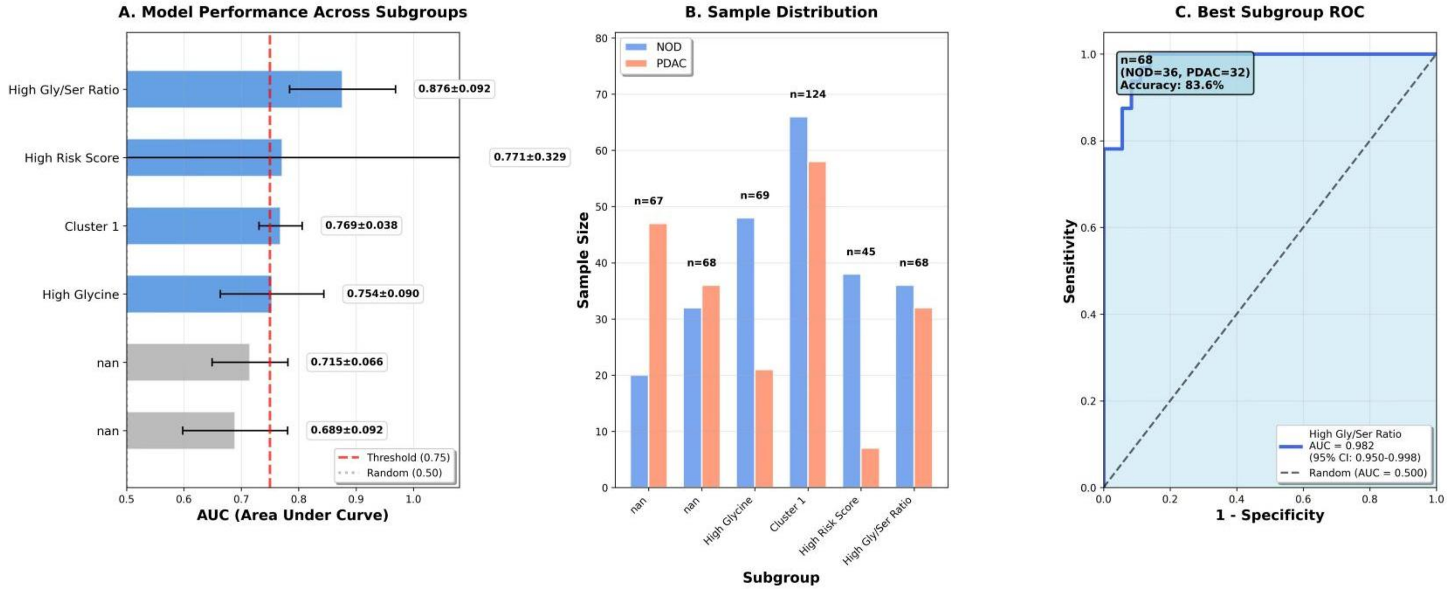

Supplementary Figure 7. Subgroup analysis of machine learning model performance

Three-panel figure demonstrating subgroup-specific model performance. (A) AUC comparison across four patient subgroups stratified by glycine/serine ratio quartiles. The high glycine/serine ratio subgroup (Q4, n=68) demonstrated superior performance (AUC=0.876, 95% CI: 0.792-0.941) compared to the overall cohort (AUC=0.853). (B) Sample distribution showing the allocation of NOD and PDAC+NOD patients across the four subgroups, confirming balanced representation. (C) ROC curve for the best-performing subgroup (high glycine/serine ratio), achieving accuracy of 83.6%, sensitivity of 82.4%, and specificity of 84.8%. This analysis suggests that the glycine/serine ratio may serve as a valuable stratification biomarker to identify NOD patients most likely to benefit from metabolite-based PDAC screening.

Supplementary Methods

1. Instrument Configuration and Analytical Methods

This study employed a Thermo Scientific Vanquish ultra-performance liquid chromatography system coupled with an Orbitrap Elite high-resolution mass spectrometry platform. Chromatographic separation was performed using a Waters ACQUITY UPLC BEH C18 column (2.1 × 100 mm, 1.7 μm) maintained at 45°C, with a flow rate of 0.3 mL/min and an injection volume of 5 μL. The mobile phase consisted of 0.1% formic acid in water (phase A) and 0.1% formic acid in acetonitrile (phase B), employing a gradient elution program: 2% B from 0-2 min, linearly increasing to 98% B from 2-15 min, maintaining 98% B from 15-18 min, rapidly returning to 2% B from 18-18.1 min, and equilibrating at 2% B until 20 min.

2. Sample Processing and Pre-analytical Handling

All clinical specimens were promptly snap-frozen in liquid nitrogen within 30 minutes of collection and stored at -80°C until analysis. Each sample underwent only a single freeze-thaw cycle prior to processing to preserve metabolite integrity. For metabolomic analysis, serum samples (100 μL) were mixed with 400 μL methanol containing internal standard (fenclonine, 2.9 g/L), followed by centrifugation at 12,000 r/min for 15 min at 4°C. The supernatant (200 μL) was transferred for UPLC-MS analysis.

3. Quality Control and Analytical Validation

To minimize analytical bias, the injection order of all samples was fully randomized across the three analytical batches conducted over a two-week period. A comprehensive quality control strategy was implemented using pooled QC samples prepared by combining equal aliquots (5 μL) from each individual sample extract, followed by thorough vortex mixing for 2 minutes. These representative QC samples were analyzed after every six experimental samples to monitor instrument stability and analytical reproducibility.

4. Data Processing and Normalization

Raw data were processed using Compound Discoverer 3.0 software for peak detection, retention time alignment, and metabolite identification. Data normalization was performed using a two-step approach: total area normalization followed by probabilistic quotient normalization. All endogenous metabolites were detected in every sample; therefore, no missing value imputation was required. Batch effects were assessed via principal component analysis of QC samples, and ComBat correction was applied despite minimal observed batch effects (ΔAUC = 0.008).

5. Quality Control Criteria

The QC acceptance criteria included: retention time drift < 0.1 min, coefficient of variation for target metabolite peak areas < 15%, mass accuracy error < 5 ppm, and signal intensity relative standard deviation < 15%. Any analytical batch with >20% of QC samples failing these criteria would require re-analysis; all batches in this study met the quality standards.

This comprehensive methodological description ensures complete traceability of experimental procedures and enhances the reproducibility and reliability of the analytical results. The dataset provided in this study can be found in online databases (Cheng, Zhiyuan (2025), "noddac", Mendeley Data, V1, doi: 10.17632/mvcgk88rmg. 1).
